# The Portfolio Pathway to Specialist Registration; Success in CESR for Applicants and Trusts

**DOI:** 10.1192/bjo.2024.302

**Published:** 2024-08-01

**Authors:** Sarah Holmes, Neeraj Berry

**Affiliations:** 1 RCPsych, Cardiff, United Kingdom; 2 RCPsych, London, United Kingdom

## Abstract

**Aims:**

In line with the 2023 legislative change and move to the New Standard of CESR, this will be an informative and educational presentation directed at CESR applicants, and Local Trusts who wish to implement support for CESR. With this suite of support we aim to dispel concerns relating to CESR.

**Methods:**

Mapping guidance to the New Standard of CESR. Production of College guidance in line with the legislative changes, to support success in CESR. Building a CESR Network for all stakeholders.

**Results:**

Delivery of training, a suite of guidance and CESR Roadshows across the four nations.

**Conclusion:**

Creating awareness and spreading communication. Ongoing support for Applicants, Trusts and other CESR stakeholders. Clarity for Applicants, particularly in relation to which Cohort to select, what evidence to include and how to submit a successful application first time.

